# Contour changes of peri-implant tissues are minimal and similar for a one- and a two-piece implant system over 12 years

**DOI:** 10.1007/s00784-020-03638-1

**Published:** 2020-10-15

**Authors:** Miha Pirc, Oliver Harbeck, Vitor M. Sapata, Jürg Hüsler, Ronald E. Jung, Christoph H. F. Hämmerle, Daniel S. Thoma

**Affiliations:** 1 Clinic of Reconstructive Dentistry, Center of Dental Medicine, University of Zurich, Plattenstrasse 11, CH-8032 Zurich, Switzerland; 2 Department of Oral Medicine and Periodontology, Faculty of Medicine, University of Ljubljana, Ljubljana, Slovenia; 3 Discipline of Periodontics, School of Dentistry, University of São Paulo, Sao Paulo, Brazil; 4 Department of Periodontology, Research Institute for Periodontal Regeneration, College of Dentistry, Yonsei University, Seoul, Korea

**Keywords:** Dental implants, Fixed, Partial, Denture, Humans

## Abstract

**Objectives:**

To assess contour changes of peri-implant tissues comparing a one- and a two-piece dental implant system over 12 years.

**Materials and methods:**

Patients seeking implant therapy were enrolled and randomly allocated to receive implants (a one-piece (STM) or a two-piece (BRA) system). Impressions were taken at the time of insertion of the final reconstruction (BL), after 1 year (FU-1), 5 years (FU-5), and at 12 years (FU-12). Thirty patients were included in the analysis (STM, 16; BRA, 14). Digital scans of casts were superimposed and analyzed in an image analysis program. Measurements included changes of the crown height, contour changes on the buccal side of the implants and the contralateral teeth (control).

**Results:**

Contour changes at implant sites revealed a loss of − 0.29 mm (STM) and − 0.46 mm (BRA) during an observation period of 12 years. Contour changes at the corresponding tooth sites amounted to − 0.06 mm (STM) and − 0.12 mm (BRA) during the same time period. The implant crown gained 0.25 mm (STM) and 0.08 mm (BRA) in height due to recession of the marginal mucosa. The corresponding gain in crown height at the contralateral tooth sites amounted to 0.36 mm (STM) and 0.10 mm (BRA). Interproximal marginal bone level changes measured − 0.28 mm (STM) and − 1.11 mm (BRA). The mean BOP amounted to 38.8% (STM) and 48.7% (BRA) at the 12-year follow-up (FU-12).

**Conclusion:**

Minimal changes of the peri-implant soft tissue contour were observed at implant sites over the period of 12 years irrespective of the use of a one- or a two-piece implant system. The differences between the implant sites and corresponding teeth were clinically negligible.

**Clinical relevance:**

Peri-implant soft tissue stability is of high clinical relevance when monitoring dental implant sites on the long run. Clinical data on the extent of soft tissue changes around different implant systems are scarce. The present RCTs demonstrate minimal changes of the peri-implant soft tissue contour 12 years after implant insertion independent of the use of a one- or a two-piece implant system.

## Introduction

Implant therapy is considered a dental treatment option with high predictability and survival rates of implants and corresponding reconstructions [1–3]. Due to the lack of clinically relevant parameters, survival rates are not sufficient from a clinicians’ perspective. Therefore, success criteria were defined adding technical, biological, and esthetic outcomes to the simple survival rates [1, 4].

Among these implant success parameters, the stability of the peri-implant hard and soft tissues appears to be relevant not only from an esthetic point of view but also from a biological point of view. Peri-implant tissue stability at the buccal aspect can be assessed applying various methods, tools, and outcome measures [5]. In the past, peri-apical X-rays and, more recently, cone-beam computed tomography were used to evaluate changes of the peri-implant tissues on the level of the bone [6, 7]. These data are limited by the fact that the clinical relevance of having a sufficient buccal hard tissue is still questionable. In fact, data based on immediate implants demonstrated that even in the case of missing buccal bone, the peri-implant soft tissues remained stable to a great extent [8, 9]. In addition, monitoring patients/implants with three-dimensional X-rays may not be ethical, as there is no “safe” dose of radiation [6]. In radiation protection, ALARA (i.e., “As Low As Reasonably Achievable”) is a fundamental principle for diagnostic radiology in medicine and dentistry [10, 11]. For selection and justification of the optimal imaging modality, ALARA was adapted to ALADA (i.e., “As Low As Diagnostically Acceptable”) [6, 12]. In order to overcome these limitations, noninvasive methods were developed to monitor implant sites over time.

Contour changes of the dentition and the implant sites can be assessed based on conventional or digital impressions. Scans of various time points are superimposed and contour changes of the peri-implant tissues calculated [13–16].

Among the confounding factors, potentially influencing the maintenance of peri-implant tissues are the design and type of the implant [17, 18]. Historically, one- and two-piece dental implant systems can be differentiated. Two-piece dental implants were designed to be placed in a two-stage procedure [19]. In contrast, the one-piece implant system consists of an endosseous and a transmucosal part emerging through the mucosa [20]. These implants are typically placed in non-submerged manner. In the literature, survival rates and marginal bone level changes are well documented for both implant types. Clinical studies evaluating the longer-term performance of dental implants compared to natural tooth sites assessed by contour changes are scarce though [21].

The aim of this study was, therefore, to assess contour changes of peri-implant tissues comparing a one- and two-piece dental implant systems over 12 years.

## Materials and methods

### Study design

This study was designed as a randomized controlled clinical trial and approved by the local ethics committee (KEK-ZH-Nr. 2014-0201). Sixty patients seeking dental implant therapy at the Clinic of Reconstructive Dentistry, Center of Dental Medicine, University of Zurich, Switzerland, were consecutively included. Patients were randomly allocated to either a one-piece (Straumann tissue level, Institute Straumann, Basel, Switzerland; STM) or a two-piece (Branemark MkIII or MkIV, Nobel Biocare, Zurich, Switzerland; BRA) dental implant system. The specific protocol, inclusion, and exclusion criteria were described in a previous publication [22].

In brief, all implant surgeries were performed according to the manufacturer’s guidelines. BRA dental implants were placed with the flat top at the bone crest and STM dental implants with the transition between the rough and smooth surface at the bone crest. Some implants were placed with increased sink depth due to prosthetic reasons. Where necessary, guided bone regeneration (GBR) procedures were performed either prior to or at implant placement. For ridge preservation procedures and primary and simultaneous sinus elevation procedures, xenogeneic materials were used. For horizontal bone regeneration prior to dental implant placement, autogenous bone blocks in combination with xenogeneic bone grafting materials were used. These materials were covered with xenogeneic or synthetic membranes.

The prosthetic procedures were performed according to the guidelines for each implant system. The decision between screw-retained or cemented final reconstructions was made based on the clinical situation and the clinician’s preference.

### Baseline and follow-up examinations

The insertion of the final reconstruction was chosen as baseline (BL), and patients were subsequently enrolled in an individually designed maintenance program with periodic visits at the dental hygienists.

All patients were recalled at 1 year (FU-1), at 5 years (FU-5), and at 12 years (FU-12) after delivery of the final reconstruction for follow-up examinations. The following biological, technical, and esthetic parameters were assessed at the BL and FU appointments:

- Probing depth (PD)
- Clinical attachment level (CAL)
- Plaque control record (PCR) [23]
- Bleeding on probing (BOP) [24]

### Model fabrication

Alginate impressions (Hydrogum 5 Zhermack, Padoua, Italy) were taken at BL and at all FU appointments. Dental stone type IV (GC, Fujirock EP, GC Europe, Leuven, Belgium) casts were fabricated and evaluated for irregularities such as porous areas, undefined gingival margins, broken cusps, or undefined vestibulum. Only suitable casts without irregularities were included in the analyses.

### Stereolithography image acquisition and matching of data

Cast models were scanned with a 3D scanner (Imetric 3D, Courgenay, Switzerland). STL (stereolithography) files from BL, FU-1, FU-5, and FU-12 were uploaded to an image analysis software (Swissmeda Software; Swissmeda AG, Zurich, Switzerland). Digital casts were superimposed automatically by the software and manually adjusted with the implant crown serving as the reference.

### Profilometric and image analysis

In case patients received more than one dental implant, one implant was randomly chosen for the analysis. The contralateral or adjacent natural tooth was included in the analysis serving as a control. Measurements were performed by a calibrated, blinded evaluator, with access to the STL files only. The following measurements were performed at BL, FU-1, FU-5, and FU-12:

1. Linear measurements: A longitudinal slice was selected dividing the crown mesiodistally into two equal parts. A line coinciding with the tooth axis was then drawn in the transversal images of the sections. Clinical crown height (CH) changes in an apico-coronal direction were assessed measuring the distance between the incisal edge and the mucosal/gingival margin axis. A line perpendicular to the tooth/implant axis was drawn to determine the changes of the estimated soft tissue thickness (eTT). The distance between this line and the buccal soft tissue contour was assessed at three different levels (1, 3, and 5 mm) apical to the mucosal/gingival margin.
2. Profilometric measurements: Contour changes (PC) were calculated by the software, measured in millimeters and corresponded to the mean distance between two surfaces. The region of interest was bordered by the mucosal/gingival margin at the analyzed site, the mesial, and distal line angles and extended 3–6 mm apically.

**Fig. 1:**
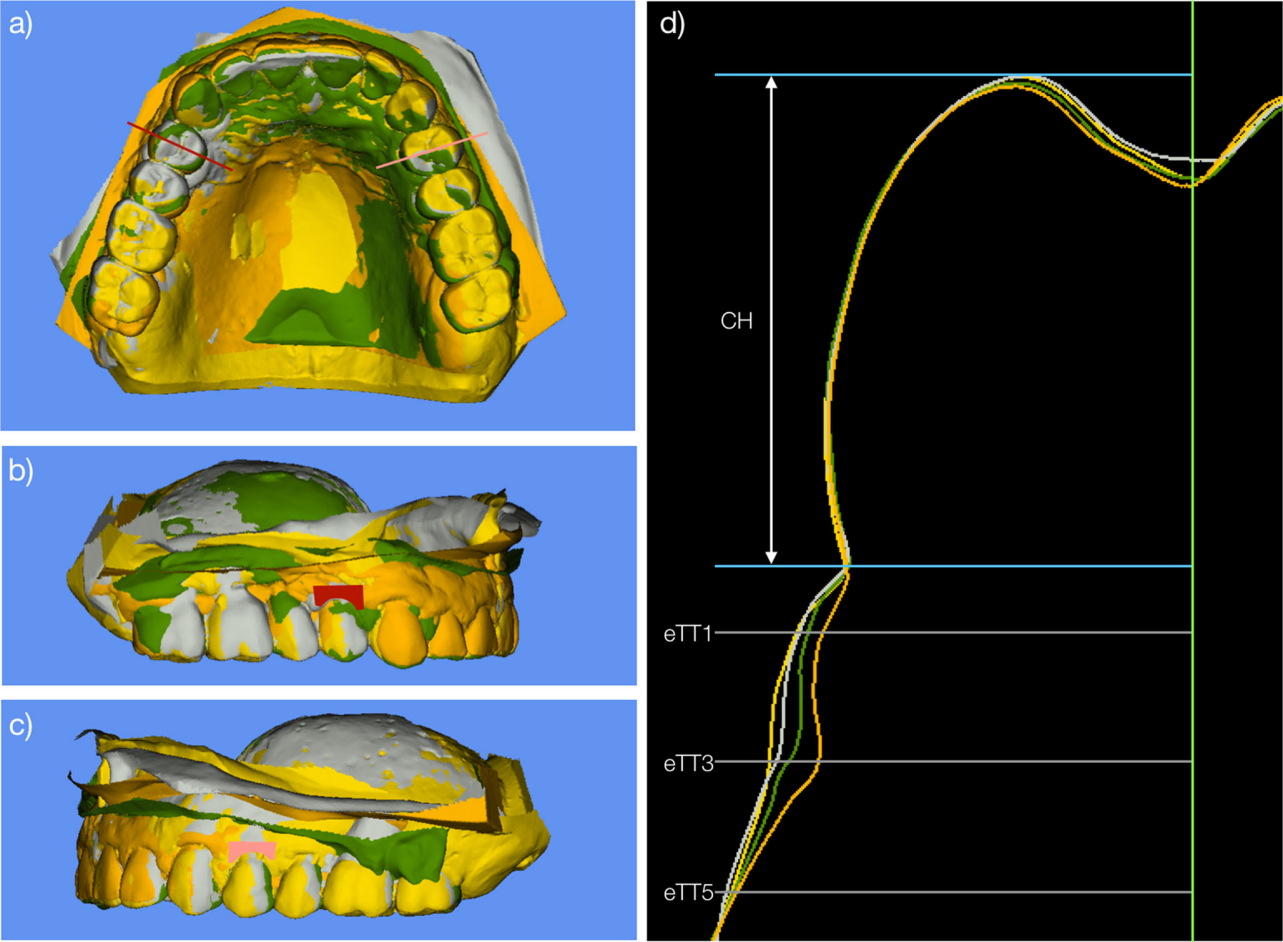
(a) Superimposed STL models. Baseline (yellow), 1-year follow-up (gray), 5-year follow-up (green), and follow-up at 12 years (orange). Red line is indicating a longitudinal slice dividing the crown mesiodistally into two equal parts. (b) Superimposed STL models with colored area (red) representing the analyzed area at the implant site. (c) Superimposed STL models with colored area (pink) representing the analyzed area at the contralateral tooth site. (d) Outline of the models and linear measurements performed in central section. CH, clinical crown height; eTT1 estimated tissue thickness at 1 mm below the gingival margin; eTT3 estimated tissue thickness at 3 mm below the gingival margin; eTT5 estimated tissue thickness at 5 mm below the gingival margin

For details, see Fig. 1.

### Radiographic measurements

Intraoral radiographs of all implants were taken at baseline (BL) and all follow-up examinations (FU) using a paralleling technique with Rinn holders. Analog films (Kodak Ekstaspeed Plus; Eastman Kodak Co., Rochester, NY, USA) were used at BL and FU-1 and were then digitized. Digital radiographs were taken at FU-5 and FU-12. Marginal bone level changes over time were calculated using an open-source software (Image J; National Institutes of Health, Bethesda, MD, USA). For calibration purposes, the known distance between two implant threads served as a reference to define the exact magnification of the images. The marginal bone level was assessed at the mesial and distal implant surfaces by measuring the distance from the reference point at the implant to the bone crest (distance implant bone, DIB). The most coronal point of the flat top of the implant (BRA) or the implant shoulder (STM) served as reference points for these measurements.

All analyses were performed by a calibrated examiner, who was not involved in the clinical procedures.

### Statistical analysis

Means and standard deviations as well as medians with quartiles were used to describe continuous variables; counts and percentages were used for categorical variables. Chi-square test was used for all categorical variables; Wilcoxon signed-rank test and Wilcoxon-Mann-Whitney test were applied on continuous variables for dependent or independent data. *P*-values ≤ 0.05 were considered statistically significant. All statistical analyses were performed with SAS 9.4 (SAS Corp., Cary NC. USA).

## Results

A total number of 60 patients (in both groups: 10 male patients and 20 female patients) were enrolled in the study. The mean age of the patients at BL was 55.7 years (SD ± 14) (BRA) and 47.8 years (SD ± 15) (STM).

At 5 years (FU-5), 33 dental casts were suitable for the profilometric analyses [25]. Out of those, 3 patients dropped out. Thirty patients (STM, 16 patients; BRA, 14 patients) were therefore included in the analyses for contour and linear changes at FU-12, with a mean follow-up time of 12.07 ± 0.73 years. The mean age of the patients at the FU-12 was 63.6 years (SD ± 15). The location of sites is summarized in Table 1.

**Table 1:** Location of the analyzed implant sites

Location	Front	Premolars	Molars
Upper jaw	6	10	2
Lower jaw		6	6
Total	6	16	8

In 24 out of 30 patients, guided bone regeneration (GBR) procedures were performed (STM, 14 patients; BRA, 10 patients). The defect configurations consisted of dehiscences ranging from 1 to 5 mm. All these defects were treated with guided bone regenerative procedures. A total of six patients with six implants did not receive any bone regenerative procedure.

The mean BOP at the implant sites was 38.8% (0; 83%) in group STM and 48.7% (17%; 83%) in group BRA at the 12-year follow-up (FU-12). The mean plaque control record (PCR) amounted to 12.3% (0; 67%) in group STM and to 12.7% (0; 83%) in group BRA at the same time point.

### Contour changes, linear measurements between baseline, and the 12-year follow-up examination

**Fig. 2:**
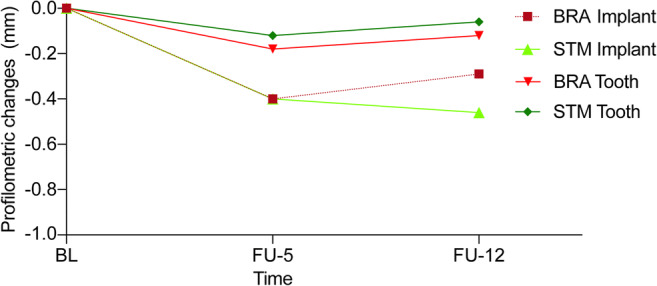
Graph representing contour changes from baseline to FU-5 and to FU-12 for implant and contralateral tooth sites

**Fig. 3:**
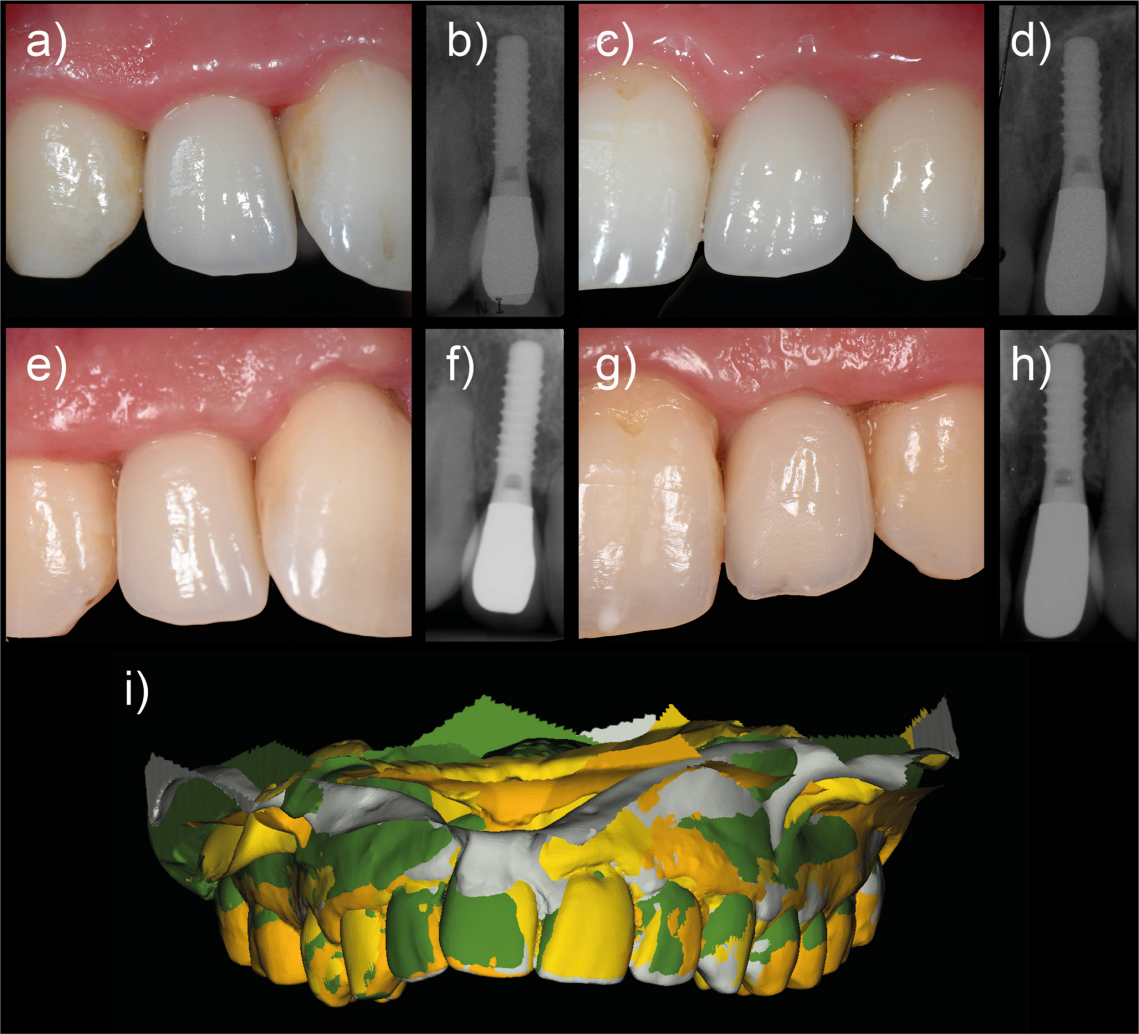
Clinical images and X-rays at different follow-up appointments: (a) right lateral incisor at 1-year follow-up, (b) X-ray at 1-year follow-up, (c) left lateral incisor at 1-year follow-up, (d) X-ray at 1-year follow-up, (e) right lateral incisor at 12-year follow-up, (f) X-ray at 12-year follow-up, (g) left lateral incisor at 12-year follow-up, (h) X-ray at 12-year follow-up, and (i) superimposition of the models

The contour changes on the buccal side of the implants (IPC BL/FU-12) revealed a difference of − 0.29 mm (− 0.52; − 0.11) (STM) and of − 0.46 mm (− 0.68; − 0.05) (BRA) (within group comparison *p* = 0.0106, *p* = 0.0029; intergroup comparison *p* = 0.787). The corresponding contour changes on the buccal side of the control teeth (TPC BL/FU-12) revealed a change of − 0.06 mm (− 0.19; 0.19) (STM) and of − 0.12 mm (− 0.28; 0.17) (BRA) (within group comparison *p* = 0.5534, *p* = 0.5016; intergroup comparison *p* = 0.755) (Figs. 2 and 3).

The differences in contour changes between implant sites and contralateral control teeth (IPC FU-12/TPC FU-12) amounted to − 0.36 mm (− 0.57, 0.03) (STM) and − 0.28 mm (− 0.87, 0) (BRA) (within group comparison *p* = 0.022, *p* = 0.017; intergroup comparison *p* = 0.755).

The median height of the implant crowns increased by 0.25 mm (− 0.05; 0.50) (STM) and 0.08 mm (− 0.54; 0.45) (BRA) from baseline to FU-12 (within group comparison *p* = 0.126, *p* = 0.670; intergroup comparison *p* = 0.244). For the corresponding control teeth, the crown height increased (TCH BL/FU-12) by 0.36 mm (0.04; 0.48) (STM) and 0.10 mm (− 0.19; 0.44) (BRA) (within group comparison *p* = 0.006, *p* = 0.334; intergroup comparison *p* = 0.244).

The difference between implants and the teeth showed the following values: the median height at FU-12 measured 0.29 mm (− 0.35, 1.11) (STM) and − 0.90 mm (− 1.39, 0.63) (BRA) (within group comparison *p* = 0.389, *p* = 0.268; intergroup comparison *p* = 0.1661).

Changes for the estimated soft tissue thickness (eTT) at implant and tooth sites are depicted in Table 2.

**Table 2:** Changes of contour, linear, and radiographic parameters between baseline and FU-12

Variables in mm (median and quartile)	BRA	STM
Implant crown height (ICH BL/FU-12)	0.08 (− 0.54; 0.45)	0.25 (− 0.05; 0.50)
Implant contour changes (IPC BL/FU-12)	− 0.29 (− 0.52;− 0.11)	− 0.46 (− 0.68; − 0.05)
Implant estimated soft tissue thickness at 1-mm IeTT1 BL/FU-12)	0.52 (0.09; 0.69)	0.60 (0.41; 1.26)
Implant estimated soft tissue thickness at 3-mm IeTT3 BL/FU-12)	0.43 (0.14; 0.74)	0.96 (0.38; 1.12)
Implant estimated soft tissue thickness at 5-mm IeTT5 BL/FU-12)	0.43 (0.09; 0.68)	0.83 (0.37; 0.98)
Distance from implant shoulder to marginal bone level (DIB BL/FU-12)	− 1.11 (− 1.56; − 0.59)	− 0.28 (− 0.75; 0.05)
Tooth crown height (TCH BL/FU-12)	0.10 (− 0.19; 0.44)	0.36 (0.04; 0.48)
Tooth contour changes (TPC BL/FU-12)	− 0.12 (− 0.28; 0.17)	− 0.06 (− 0.19; 0.19)
Tooth estimated soft tissue thickness at 1-mm TeTT1 BL/FU-12)	0.06 (− 0.19; 0.23)	0.43 (0.25; 0.60)
Tooth estimated soft tissue thickness at 3-mm TeTT3 BL/FU-12)	0.01 (− 0.32; 0.13)	0.43 (0.25; 0.55)
Tooth estimated soft tissue thickness at 5-mm TeTT5 BL/FU-12)	− 0.23 (− 0.41; − 0.14)	0.28 (0.20; 0.57)

### Contour changes and linear measurements between the 5-year and the 12-year follow-up examination

The contour changes on the buccal side of the implants (IPC FU-5/FU-12) revealed a change of − 0.04 mm (− 0.37, 0.12) (STM) and of − 0.11 mm (− 0.20, − 0.05) (BRA) (within group comparison *p* = 0.487, *p* = 0.076; intergroup comparison *p* = 0.662). The corresponding contour changes on the buccal side of the control teeth (TPC FU-5/FU-12) revealed a change of − 0.08 mm (− 0.32, 0.14) (STM) and of − 0.04 mm (− 0.24, 0.09) (BRA) (within group comparison *p* = 0.257, *p* = 0.492; intergroup comparison *p* = 0.868) (Fig. 2).

The differences in contour changes on the buccal side between the implant and contralateral control teeth (IPC FU-5/FU-12) were of 0.05 mm (− 0.44, 0.30) (STM) and − 0.03 mm (− 0.17, 0.16) (BRA) (within group comparison *p* = 0.910, *p* = 1.000; intergroup comparison *p* = 1.000).

The crown height (ICH FU-5/FU-12) for implant reconstructions increased by 0.38 mm (− 0.08; 0.98) (STM) and 0.05 mm (− 0.07; 0.21) (BRA) (within group comparison *p* = 0.039, *p* = 0.261; intergroup comparison *p* = 0.135), thereby revealing slight recessions. For the corresponding control teeth, the crown height changes (TCH FU-5/FU-12) amounted to 0.32 mm (− 0.15; 0.73) (STM) and 0.05 mm (− 0.13; 0.35) (BRA) (within group comparison *p* = 0.159, *p* = 0.340; intergroup comparison *p* = 0.299).

The change for the eTT at implant and tooth sites can be found in Table 3.

**Table 3:** Changes of contour, linear, and radiographic parameters between FU-5 and FU-12

Variables in mm (median and quartile)	BRA	STM
Implant crown height (ICH FU-5/FU-12)	0.05 (− 0.07; 0.21)	0.38 (− 0.08; 0.98)
Implant contour changes (IPC FU-5/FU-12)	− 0.11 (− 0.20, − 0.05)	− 0.04 (− 0.37, 0.12)
Implant estimated soft tissue thickness at 1-mm IeTT1 FU-5/FU-12)	− 0.21 (− 0.47; 0.20)	0.17 (0.03; 0.58)
Implant estimated soft tissue thickness at 3-mm IeTT3 FU-5/FU-12)	− 0.09 (− 0.34; 0.23)	0.29 (0.12; 0.67)
Implant estimated soft tissue thickness at 5-mm IeTT5 FU-5/FU-12)	0.00 (− 0.39; 0.27)	0.17 (0.09; 0.54)
Tooth crown height (TCH FU-5/FU-12)	0.05 (− 0.13; 0.35)	0.32 (− 0.15; 0.73)
Tooth contour changes (TPC FU-5/FU-12)	− 0.04 (− 0.24, 0.09)	− 0.08 (− 0.32, 0.14)
Tooth estimated soft tissue thickness at 1-mm TeTT1 FU-5/FU-12)	− 0.14 (− 0.31; 0.00)	0.18 (0.06; 0.62)
Tooth estimated soft tissue thickness at 3-mm TeTT3 FU-5/FU-12)	− 0.11 (− 0.37; 0.09)	0.40 (0.13; 0.57)
Tooth estimated soft tissue thickness at 5-mm TeTT5 FU-5/FU-12)	− 0.22 (− 0.60; 0.02)	0.30 (0.03; 0.46)

### Radiographic parameters

The median radiographic bone level at the 12-year follow-up (FU-12) amounted to − 0.28 mm (− 0.75; 0.05) (STM) and − 1.11 mm (− 1.56; − 0.59) (BRA) (within group comparison with median 0: *p* = 0.0386, *p* = 0.002; intergroup comparison *p* = 0.0138) (Table 2).

The median radiographic bone level changes between the baseline (BL) and the 12-year follow-up (FU-12) amounted to − 0.30 mm (− 1.13; − 0.01) (STM) and − 0.45 mm (− 0.68; − 0.15) (BRA) (within group comparison *p* = 0.0136, *p* = 0.003; intergroup comparison *p* = 0.908) (Table 3).

The median changes between FU-5 and FU-12 amounted to − 0.20 mm (− 0.84, − 0.05) (STM) and − 0.28 mm (− 0.41, − 0.03) (BRA) (within group comparison *p* = 0.002, *p* = 0.020; intergroup comparison *p* = 0.728).

## Discussion

The present RCT following patient over 12 years predominantly revealed: (i) minimal changes of the peri-implant tissue contour irrespective of the implant system; (ii) stable tissue dimensions at contralateral tooth sites; (iii) minimal recessions at both, implant and tooth sites; and (iv) minimal marginal bone loss for both implant systems, not affecting peri-implant buccal tissue dimensions.

High implant survival rates have been reported in a plethora of clinical studies resulting in confidence for this treatment modality [1, 2, 26]. Monitoring patients with dental implants is considered to be of key importance. This is predominantly due to the fact that a certain number of technical and biological complications occur over time. Apart from clinical radiographic examinations, noninvasive methods have been proposed (conventional and digital impression techniques). Among those, impressions taken at various time points during the follow-up allow assessing implant sites for changes of the peri-implant tissues. In the present study, profilometric measurements were performed over a 10- to 12-year period. The data demonstrated minimal changes of the peri-implant tissue dimension amounting to less than 0.5 mm. This is in line with the scientific literature demonstrating that the majority of the contour changes around implants occur at the early stage after placement of the final reconstructions and remain stable thereafter [27–30]. The implant site as well as the location is a factor to be considered when analyzing outcomes following implant therapy. The currently available literature reporting long-term changes of the peri-implant tissues is limited and unfortunately only reports on changes of the peri-implant tissues in the esthetic zone [31, 32]. In these regions of the jaw, changes of the peri-implant tissue remained, however, minimal, once final reconstructions were inserted [31, 32].

The true extent of peri-implant tissue changes observed can only be realized when comparisons to natural teeth are made. In the past, periodontal health has been assessed and reported at tooth sites on the long run [33, 34]. However, scientific evidence on three-dimensional changes of the periodontal tissues is limited as measurements predominantly included probing depth values, attachment loss, and the level of the margo gingivae. The present study demonstrated stable soft tissue dimensions on the buccal side of control teeth over 12 years. The comparison between the tooth and implant sites revealed slightly more stable tissue dimensions at tooth sites. The changes ranged between − 0.28 and 0.19 mm, whereas the corresponding values at implant sites ranged between − 0.68 and − 0.05 mm. This is in line with previous studies reporting a higher loss of soft tissues at implant compared to tooth sites [35, 36].

From an esthetic point of view, measurements of the implant crown height at different time points allow assessing recessions. In the present study, the observed recessions ranged between 0.08 mm (two-piece implant system) and 0.25 mm (one-piece implant system). This translates into a loss of 0.01 mm per year and 0.02 mm per year. These numbers compare well with previously reported clinical data with reported recessions ranging between 0.17 mm [32] and 0.38 mm [37]. The figures obtained at the control teeth were in the same range as at the implant sites and ranged between 0.1 mm (0.01 mm per year) and 0.36 mm (0.03 mm per year). The calculated extent of the recessions was slightly lower than in previous clinical studies [33, 34]. In these studies, the annual attachment loss ranged between 0.04 mm [33] and 0.14 mm [34].

The traditional method of monitoring dental implants and serving as one of the parameters to define between peri-implant health and disease includes the assessment of marginal bone level changes. In the present study, a one-piece and a two-piece were compared. Based on long-term clinical studies, tissue remodeling should lead to marginal bone levels located at 0.39–1.8 mm (one-piece implants) and 0.56–1.6 mm (two-piece implants) [38] below the implant shoulder. Data obtained in the present study revealed marginal bone levels being located at 1.11 mm (BRA) and 0.28 mm (STM) below the implant shoulder, thereby being well in line with the abovementioned systematic review.

There has been some speculation on the relation between the level of the interdental bone adjacent to an implant and whether this affects the stability of the buccal peri-implant tissues. When analyzing the data of the buccal tissue volumes, the majority of the loss (0.39 mm (BRA and 0.40 mm (STM)) occurred within the first 5 years after insertion of final reconstructions. The same applies to marginal bone levels that underwent a major remodeling during the same period [25]. Thereafter, between 5 years and the 12-year follow-up, changes were minimal and similar to previously published data [27, 28].

From a scientific point of view, the stability of the buccal tissues depends on the stability of the underlying hard and soft tissues. Limited by the lack of CBCT data that would reveal the full extent of the buccal bone and its relative changes over time, contour changes cannot explain the relationship between hard and soft tissues. Based on recent scientific evidence, soft tissues appear to be more stable than hard tissues at single implant sites [8, 9, 39, 40]. This is underlined by an increasing soft tissue thickness but stable peri-implant tissue dimensions [40] and soft tissues that might compensate for missing buccal bone [9]. There can, though, only be speculation what happened between the implant surface and the buccal tissue contour in the present study since no CBCT data were obtained.

## Conclusions

Minimal changes of the peri-implant soft tissue contour were observed at implant sites over the observation period of 12 years and irrespective of the implant system. The differences between the implant sites and the corresponding natural tooth sites were negligible and without clinical relevance. Marginal bone levels revealed a slightly higher loss for two-piece implants compared to one-piece implants without affecting the peri-implant contour.
